# Zika Virus Epidemic in Brazil. II. Post-Mortem Analyses of Neonates with Microcephaly, Stillbirths, and Miscarriage

**DOI:** 10.3390/jcm7120496

**Published:** 2018-11-28

**Authors:** Raimunda S. S. Azevedo, Marialva T. Araujo, Consuelo S. Oliveira, Arnaldo Jorge Martins Filho, Bruno T. D. Nunes, Daniele F. Henriques, Eliana V. P. Silva, Valéria L. Carvalho, Jannifer O. Chiang, Lívia C. Martins, Barbara C. B. Vasconcelos, Jorge R. Sousa, Fernanda Montenegro C. Araujo, Erlane M. Ribeiro, Andrezza R. P. Castro, Maria G. L. de Queiroz, Mariana P. Verotti, Márcio R. T. Nunes, Ana C. R. Cruz, Sueli G. Rodrigues, Pei-Yong Shi, Juarez A. S. Quaresma, Robert B. Tesh, Pedro F. C. Vasconcelos

**Affiliations:** 1 Seção de Arbovirologia e Febres Hemorrágicas, Instituto Evandro Chagas, Ministério da Saúde, Rodovia BR-316, km-07, Ananindeua 67030-000, Brazil; raimundaazevedo@iec.gov.br (R.S.S.A.); marialvaaraujo@iec.gov.br (M.T.A.); consuelooliveira@iec.gov.br (C.S.O.); arnaldofilho@iec.gov.br (A.J.M.F.); brunonunes@iec.gov.br (B.T.D.N.); danielehenriques@iec.gov.br (D.F.H.); elianapinto@iec.gov.br (E.V.P.S.); valeriacarvalho@iec.gov.br (V.L.C.); janniferchiang@iec.gov.br (J.O.C.); liviamartins@iec.gov.br (L.C.M.); krekrodrigues@gmail.com (J.R.S.); marcionunesbrasil@yahoo.com.br (M.R.T.N.); anacecilia@iec.gov.br (A.C.R.C.); suelirodrigues@iec.gov.br (S.G.R.); 2 Centro de Ciências Biológicas e da Saúde, Universidade do Estado do Pará, Travessa Perebebuí, 2623, Belém 66087-670, Brazil; barbaravasconcelos@globo.com (B.C.B.V.); juarez.quaresma@gmail.com (J.A.S.Q.); 3 Programa de Pós-graduação em Doenças Tropicais, Universidade Federal do Pará, Avenida Generalíssimo Deodoro 92, Belém 66055-240, Brazil; 4 Laboratório Central, Secretaria de Saúde Pública do Ceará, Avenida Barão de Studart 2405, Fortaleza 60120-002, Brazil; fernandamontenegrocaraujo@gmail.com; 5 Hospital Geral César Cals, Secretaria de Saúde Pública do Ceará, Avenida Imperador 545, Fortaleza 60015-152, Brazil; erlaneribeiro@yahoo.com.br (E.M.R.); andrezzaporto@hotmail.com (A.R.P.C.); 6 Laboratório Central, Secretaria de Saúde Pública do Rio Grande do Norte, Rua Cônego Monte 410, Natal 59037-170, Brazil; lacenrn@yahoo.com.br; 7 Fundação para o Desenvolvimento Científico e Tecnológico em Saúde (FIOTEC), Fundação Osvaldo Cruz (Fiocruz), SMPW Quadra 4 Conj. 1 Lote 3 Unidade E, Distrito Federal, Brasília 71735-401, Brazil; mpastorelloverotti@gmail.com; 8 Department of Pathology, University of Texas Medical Branch, 301 University Boulevard, Galveston, TX 77555-0144, USA; peshi@utmb.edu (P.Y.S.); rtesh@utmb.edu (R.B.T.)

**Keywords:** Zika virus, microcephaly, congenital syndrome, ZIKV RNA, ZIKV antigen

## Abstract

Introduction: The recent Zika virus(ZIKV) epidemic in Brazil was characterized by a range of different clinical presentations, particularly microcephaly, Guillain-Barré syndrome, and death. In this context, we determined the causal relationship between fatal microcephaly cases and ZIKV infection. Methods: Twelve fatal cases of neonates, whose mothers were infected with ZIKV during pregnancy, were examined; cases included nine neonatal deaths due to microcephaly, one miscarriage, and two stillbirths. Tissue samples were obtained from all cases at necropsy and were submitted for virological investigation (RT-qPCR and virus isolation) and/or histopathology (hematoxylin and eosin staining) and immunohistochemical assay for the detection of ZIKV antigens. Results: ZIKV antigens and/or ZIKV RNA were detected in tissue samples of all 12 cases examined. ZIKV was recovered in one case. Results of the virological and immunohistochemical analyses, as well as the anatomic abnormalities and histopathologic changes observed at necropsy on the 12 fatal cases, are presented. Conclusions: Data from these 12 cases provide strong evidence of the causal relationship between ZIKV and congenital disease in fetuses of women who were infected with the virus during pregnancy.

## 1. Introduction

Zika virus (ZIKV), a mosquito-borne agent belonging to the genus *Flavivirus* family *Flaviviridae*, was originally isolated in Uganda in 1947 [1]. Subsequently, ZIKV has spread globally [2]. In 2013, the virus was associated with a large epidemic in French Polynesia [3]; and since 2015, 47 countries and territories have confirmed the local transmission of ZIKV disease. Further, 27 countries and territories in the Americas have reported confirmed cases of congenital disease associated with ZIKV infection [4]. In 2015, ZIKV infections were detected in the Brazilian state of Bahia [5,6]. However, a later study reported that the introduction of the virus into Brazil actually occurred in 2013, suggesting the failure of early recognition contributed to the extensive and silent spread of the virus throughout the country [7].

The ZIKV epidemic in Brazil was characterized by a range of different clinical presentations, particularly microcephaly, Guillain-Barré syndrome, and death in individuals with autoimmune disorders [8]. The Brazilian Ministry of Health and the Pan American Health Organization (PAHO) confirmed the causal relationship between microcephaly and ZIKV after diagnosis at the Evandro Chagas Institute (Ministry of Health) of a case of microcephaly and other neurological manifestations in a newborn that died 5 min after birth. In this case, ZIKV was isolated from the brain and viral RNA was detected by real-time RT-PCR in the brain and other organs, including lungs, heart, liver and kidney [7,9]. In the present article virological, histological and immunohistochemical studies were done on 12 fatal cases, including nine newborn cases of microcephaly, one miscarriage, and two stillbirths caused by ZIKV infection. The purpose of this report is to use these confirmed cases of fatal microcephaly to demonstrate the clinical, virological, and histopathological findings associated with ZIKV infection.

## 2. Material and Methods

### 2.1. Patients

The study included 12 cases of fatal ZIKV infection that occurred between November 2015 and January 2016 from the northeast region of Brazil (states of Ceará-CE (*n* = 5), Paraíba-PB (*n* = 1), Pernambuco-PE (*n* = 1), and Rio Grande do Norte-RN (*n* = 4)), as well as from the southeast (state of Espírito Santo-ES (*n* = 1)). Table 1 summarizes the demographic and clinical characteristics and Table 2 gives the laboratory and necropsy findings on the 12 cases.

Microcephaly was diagnosed in the cases investigated after stillbirth (*n* = 2) and in newborns that had died after delivery (*n* = 9). An additional case of abortion was also included in the study, totaling 12 cases. The cases described are shown in Table 1.

### 2.2. Ethics Statement

Biological samples of the patients were obtained and processed in the context of the emergency definition by the Ministry of Health during surveillance activities of the ZIKV epidemic in Brazil. This study was approved (opinion number 1.888.946) by the Research Ethics Committee (CEP) of the Evandro Chagas Institute (IEC). Photograph of case 10 was authorized by parents (See authorization as appendix).

### 2.3. Real Time RT-PCR (RT-qPCR) and Virus Isolation

Attempts to isolate virus were performed using C6/36 cell cultures [10]. Briefly, blood and tissue fragments were triturated in phosphate-buffered saline containing 10% fetal bovine serum and antibiotics. After centrifugation at 2100× *g* for 10 min at 4 °C, 100 µL of the supernatant was inoculated into a 25-cm^2^ tissue culture flask with a monolayer of mosquito cells. After incubation for 2 h at 28 °C, medium was added and the cells were incubated at room temperature (~25 °C) for 10 days. The cells were first examined by an indirect immunofluorescent assay using a flavivirus group hyperimmune polyclonal mouse antibody. Next, cells that did not react to dengue and yellow fever virus monoclonal antibodies produced by Biomanguinhos/FIOCRUZ (DENV1-041118FDEN1P, DENV2-070109FDEN2P, DENV3-070910FDEN3P, 041005FDEN4P and foy YFV-111108FFAD2PB lots, Biomanguinhos/FIOCRUZ, Rio de Janeiro, Brazil) were tested by real-time RT-PCR (RT-qPCR) [11,12]. Supernatants of tissue homogenates and, when available serum samples, were used for RNA extraction with the Trizol Plus RNA Extraction kit (Ambion, Thermo-FischerScientific, Waltham, MA, USA), according to manufacturer instructions. The RT-qPCR assays were carried out in a 7500 Real Time PCR System (Applied Biotechnologies, Thermo-Fischer Scientific, Waltham, MA, USA) using the Superscript III Platinum One-Step RT-qPCR kit (Invitrogen, Thermo-Fischer Scientific, Waltham, MA, USA) and two different primer/probe sets targeting the NS5 and E regions of the ZIKV genome.All clinical samples were simultaneously tested by RT-qPCR against dengue virus (DENV) and chikungunya virus.

### 2.4. Histopathology and Immunohistochemistry

Paraffin-embedded tissue samples were processed for histopathology and stained with hematoxylin and eosin (HE). For immunohistochemistry (IHC), an adapted Streptavidin Alkaline Phosphatase (SAAP) assay [8,13,14,15,16,17,18] with anti-ZIKV polyclonal mouse antibody. The polyclonal anti-ZIKV antibody used in the IHC assay was prepared at the Instituto Evandro Chagas (IEC, Ananindeua, Brazil) in young Swiss mice using a ZIKV strain isolated in cell culture (C6/36 cells). This polyclonal serum was tested against antigens of ZIKV and several other circulating flaviviruses (dengue virus—DENV, yellow fever virus–YFV, Saint Louis encephalitis virus—SLEV, etc.) in Brazil and was only positive for ZIKV. All clinical samples were simultaneously tested by IHC against ZIKV and the most important flaviviruses occurring in Brazil (DENV and Yellow fever virus-YFV) using monoclonal antibodies.

## 3. Results

Table 2 shows the results of the RT-qPCR, histopathology, and immunohistochemistry assays for ZIKV in tissues taken at necropsy from the 12 cases. A single ZIKV strain was isolated from the brain, heart, kidney, and lungs of Case 10. The clinical specimens were also subjected to RT-qPCR for the detection of dengue and chikungunya, and all of the specimens tested negative. In addition, immunohistochemistry assays for dengue, yellow fever, and chikungunya viruses were also negative in all examined clinical specimens. In contrast, ZIKV antigens were detected in the tissues of all 12 cases, with the brain and liver tissues showing the strongest positive (and ZIKV load titers) results (Table 2).

**Figure 1:**
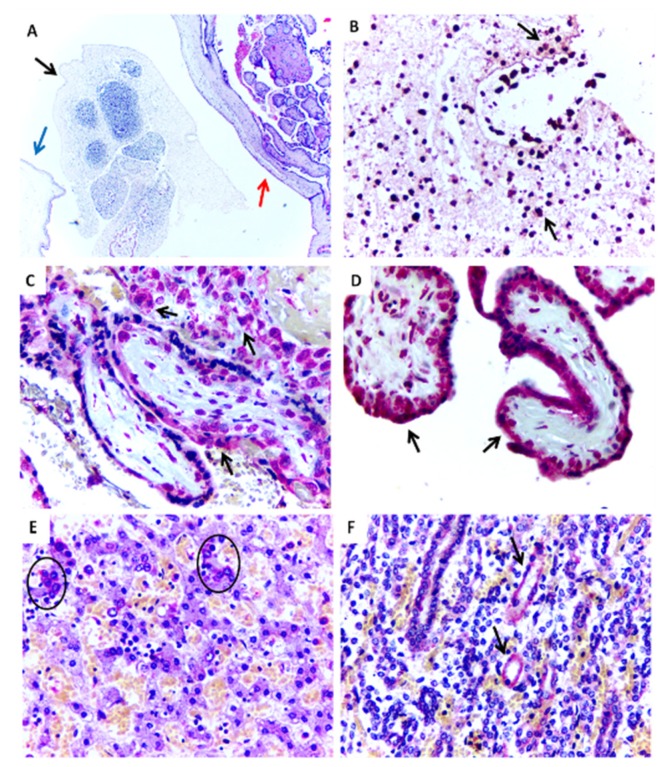
Histopathological aspects and positive immunostaining of the ZIKV-infectedmiscarriage (Case 1). (**A**) Embryonic tissue (black arrow) within the yolk sac (blue arrow) and placenta (red arrow) (HE, 100×). (**B**) Positive immunostaining for ZIKV antigens in embryonic tissue (IHC, SAAP, 400×). (**C**,**D**) Positive immunostaining for ZIKV in placental chorionic villi (IHC, SAAP, 400×). (**E**) Positive immunostaining to ZIKV observed in the liver in hepatocytes (circle) (Case 3) (IHC, SAAP, 400×). (**F**) Positive immunostaining to ZIKV observed in the kidney in renal tubule (black arrows) (Case 3) (IHC, SAAP, 400×).

Case 1 showed necrotic lesions in fetal tissues and calcifications in the placenta. ZIKV antigens were detected in both tissues (Figure 1A–D). Fresh tissues were not obtained.

Case 2 showed large areas of cerebral necrosis, apoptosis, focal areas with calcifications, gliosis, vascular proliferation, and edema. ZIKV antigens, detected by immunohistochemistry and viral RNA, were found in all tissues studied (brain, liver, kidney, and lungs) (Table 2). In Case 3, histopathology revealed apoptotic neurons, diffuse vascular congestion and edema in the brain. Similarly, alterations were seen in the liver (congestion and steatosis), placenta (multifocal calcification areas), and lungs (congestion). Immunohistochemistry was ZIKV-positive only in the brain, liver, and kidney (Table 2) (Figure 1E,F).

Amniotic fluid and meconium were found in the lungs, strongly suggesting fetal distress and aspiration. Fresh tissues were not obtained (Table 2). Histopathological changes were observed in almost all examined tissues. The central nervous system (CNS) was the most affected tissue, but several changes were also found in sections of viscera, mainly liver, kidney, and lungs (Table 2).

**Figure 2:**
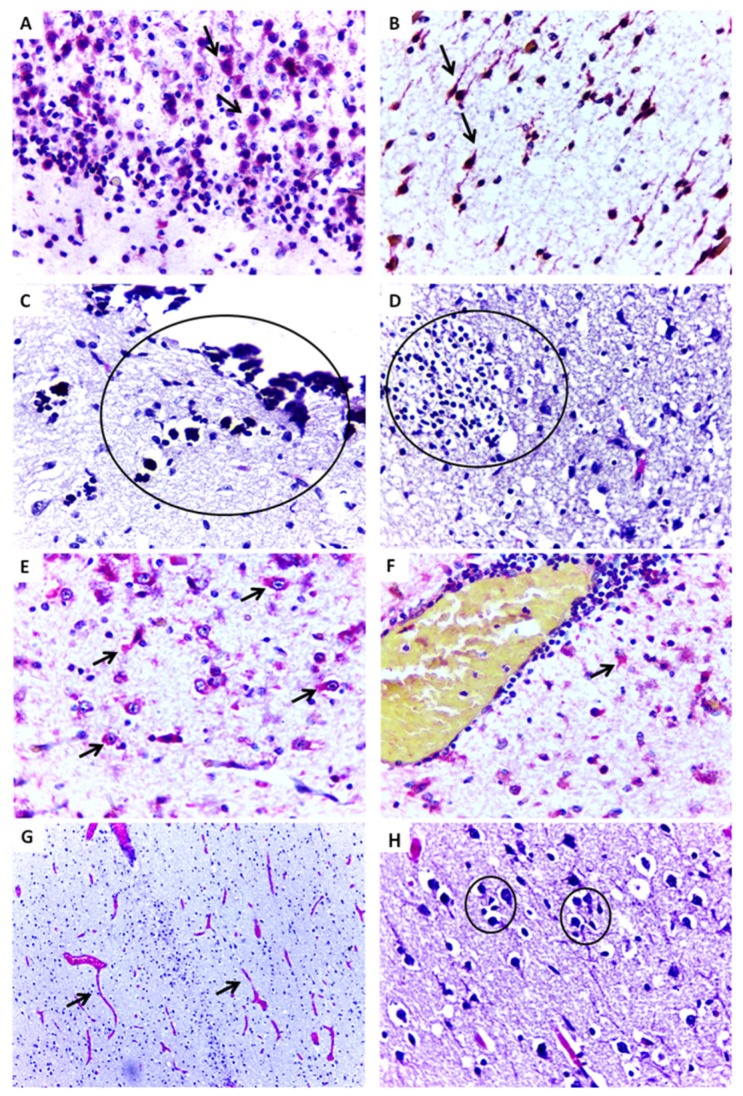
Representative histopathological changes and positive immunostaining in central nervous tissue of ZIKV-positive microcephaly cases by ZIKV. (**A**,**B**) Cellular disorganization in the cortical layer and positive immunostaining (arrow) for ZIKV antigen in neurons (Case 4) (IHC, SAAP, 400×). (**C**) Foci of dystrophic calcification in parenchyma with degenerative necrotic lesion (Case 6) (HE, 400×). (**D**) Gliosis in parenchyma with degenerative necrotic lesion (Case 6) (HE, 400×). (**E**,**F**) Positive immunostaining for ZIKV in neurons and astrocytes with perivascular inflammatory infiltrate (Case 6) (IHC, SAAP, 400×). (**G**) Significant vascular proliferation, edema and vessel congestion (arrows) (Case 11) (HE, 100×). (**H**) Pyramidal neurons of the cerebral cortex with neuronophagy, satelitosis, and gliosis (circles) (Case 11) (HE, 400×).

Case 4 only survived for 4 hbut had microcephaly and other malformations (nasopalatine fissure, low set ears, and congenital clubfoot). Vascular congestion was observed in all examined tissues (brain, liver, kidney, and lungs). Edema in the brain, focal areas of steatosis in the liver, and focal hemorrhage in the kidney and lungs were frequently found. Immunohistochemistry exams were positive in the brain (Figure 2A,B) and other tissues, except for the lungs (Table 2).

Case 5 survived for 6 hand had microcephaly, arthrogryposis, and other malformations. The mother had a confirmed ZIKV infection during pregnancy; and brain fragments of the infant tested positive for the virus by RT-qPCR (RNA detection) and immunohistochemistry (antigen detection). In addition to relevant histopathological findings observed in the brainstem (edema, vascular congestion, and degenerative neuronal lesions), significant changes were found in the liver (subcapsular bleeding, steatosis, and inflammatory infiltrate), kidney, and lungs (focal hemorrhage and vascular congestion) (Table 2).

Case 6 had microcephaly, arthrogryposis, other congenital malformations and survived for 14 h. Histopathological analysis revealed intense brain damage characterized by necrosis, apoptosis, gliosis, calcifications, ventriculomegaly, edema, intense vascular congestion, and inflammatory infiltration (Figure 2C,D). Like the other cases, vascular congestion was observed in the liver, kidney, and lungs, as well as hepatic steatosis, pulmonary bleeding, and edema. Immunohistochemistry was positive for ZIKV antigen (Figure 2E,F) and viral RNA was detected by RT-qPCR in the brain (Table 2).

Case 7 died 1 day after birth. Intense histological changes were observed in neuronal tissue, including meningeal thickening, as well as edema, hyperemia, intense vascularization, and an inflammatory infiltrate in all CNS structures examined. Intense vascular proliferation and focal necrotic lesions were frequent. In the lung, an inflammatory infiltrate mainly consisting of lymphocytes was detected in the bronchioles and interstitial septa, accompanied by alveolar collapse. Immunohistochemistry was only positive for ZIKV antigen in the brain, while the ZIKV genome was detected in the brain, liver, spleen, heart, kidney, and lungs (Table 2).

Case 8 survived for 2 days and had microcephaly. Histological changes were mainly found in the CNS and included intense vascular congestion, edema, vascular proliferation, neuronal necrosis, focal neuronal depopulation, and multiple calcification foci. Aspiration of amniotic fluid was observed in the lungs, suggesting fetal distress. Immunohistochemistry was positive for ZIKV antigens in all tissues examined (Table 2).

Case 9 survived for 1 day and had microcephaly and other severe congenital malformations (congenital clubfoot and agenesis of the fingers). In the brain, multiple areas of cortical calcifications were observed, as well as neuronal depopulation, edema, necrosis, focal gliosis, vascular proliferation and residual necrotic neurons. Immunohistochemistry was positive in the brain, liver, and kidney. ZIKV RNA was detected in the brain, kidney, and lungs (Table 2).

**Figure 3:**
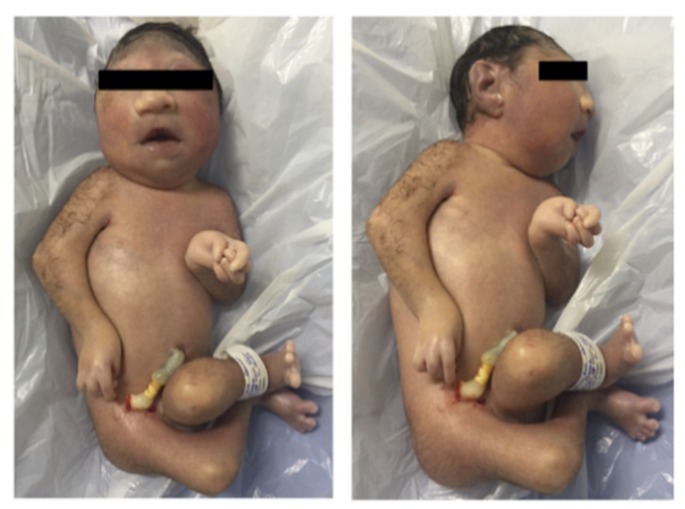
Newborn with 29 weeks gestational age showing microcephaly and arthrogryposis. The infantdied 5 min after birth (Case 10).

Case 10 (Figure 3) survived for only 5 min and had microcephaly, arthrogryposis and other congenital malformations includingatresia of esophagus and insipient trachea. ZIKV RNA was detected by RT-qPCR in cerebrospinal fluid, brain, heart, kidney, and lungs. ZIKV was isolated in C6/36 cells from the brain, heart, kidney, and lungs. Formalin-fixed samples were not obtained (Table 2).

Case 11 survived for 1 day and had microcephaly and arthrogryposis. Important changes were observed in CNS tissues, including meningeal thickening, intense vascular proliferation, edema, neuronal destruction followed by depopulation, and focal gliosis in the cortex (Figure 2G,H). Viral RNA was detected in all tissue samples (brain, liver, spleen, heart and kidney), and immunohistochemistry identified ZIKV antigens in the brain, liver, heart and kidney (Table 2).

Case 12 survived for 1 day. Placental tissue contained several foci ofcalcification and immunohistochemistry was positive for ZIKV antigens. ZIKV RNA was detected by real-time PCR in the placenta and in a neonatal serum sample. Fresh and formalin-fixed tissues samples were not collected from this case (Table 2).

## 4. Discussion

Until recently, ZIKV was not considered a serious threat or important public health problem. However, with the onset of Zika fever outbreaks and the microcephaly epidemic in Brazil and other countries, reports of microcephaly and other congenital malformations started to appear with cases of ZIKV infection, and the term “congenital ZIKV syndrome” was proposed [19,20,21,22]. In view of the severity of some cases with intense brain damage and other malformations, the Brazilian Government, and thereafter the WHO, classified the ZIKV epidemic as a public health emergency of international concern [23].

The first link between ZIKV and microcephaly in Brazil was established at the Evandro Chagas Institute with the detection of the ZIKV genome in the brain and other visceral fragmentsof a fatal case [9], and later in follow-ups of pregnant women infected with ZIKV in Rio de Janeiro [24,25]. These reports and others [21,26,27] demonstrated the need for clinical and laboratory evaluation of as many microcephaly cases as possible. Neonates who have died with microcephaly shouldbe examined to demonstrate the presence of ZIKV antigens by immunohistochemistry in paraffin-embedded tissues, or of ZIKV RNA by RT-qPCR in fresh tissues, cerebrospinal fluid, and serum samples, as previously reported [7,8,27,28].

In the present study, all 12 cases presented with severe and intense CNS involvement, which was characterized by necrosis, apoptosis, diffuse vascular congestion and proliferation, edema, inflammatory infiltration, gliosis, and calcifications. The predominance of lesions typical of neuronal tissue destruction is almost pathognomonic of the congenital ZIKV syndrome. In almost all of the cases, neuronal destruction (depopulation) was observed, which was well illustrated in our Cases 8, 9 and 11 of microcephaly.

In Case 1, ZIKV antigens were detected in embryonic tissues, neuronal cells, hepatocytes, and tubular renal cells. This case was a miscarriage that occurred at 8 weeks of gestation, demonstrating the destructive potential of ZIKV and characterizing the intense neurotropism of the virus. In addition, these findings emphasize the devastating fetal CNS injury following infection of the mother with ZIKV during pregnancy.

Important alterations were also detected in hepatic tissues, including steatosis, vascular congestion, focal erythropoiesis, and inflammatory infiltrates, similar to the descriptions of changes seen with other arboviruses, such as yellow fever and dengue virus [29,30,31]. In addition, renal changes were also found in our cases, especially vascular congestion, a finding also reported with dengue virus infection [32]. ZIKV RNA and/or antigens were detected in all cases, even in the child that died only 5 min after birth, as well as in the cases of microcephaly, stillbirth, and miscarriage, whose mothers were infected in the first or second trimester of gestation. These findings reinforce the capacity of ZIKV to persist in tissues of miscarriages, stillbirths, and neonates.

This case series demonstrates the potential of ZIKV to cause intrauterine infection, resultingine death at birth. The CNS lesions observed in our cases of miscarriage, stillbirth and newborn infections were predominantly characterized by neuronal death (necrosis and apoptosis), gliosis and calcifications;whereas lesions observed in the fatal adult cases have been predominantly inflammatory [8,13,14,15,16,17]. In conclusion, this study provides important new information on the relationship of ZIKV infection with microcephaly and other congenital defects in cases of congenital ZIKV syndrome. The detection of ZIKV antigens and viral RNA in CNS cells, especially neurons, and also in other cells of viscera in fetal and neonatal cases suggests that ZIKV persists in these cells. In our case series, the presence of ZIKV antigens and/or RNA in the 12 cases in Brazil was confirmed, indicating the causative role of ZIKV in nine neonate deaths, two stillbirths, and one miscarriage (ZIKV detected in placenta). In addition, cases 10 and 12 demonstrate that congenital ZIKV infection persisted for several months since the motherswere infected in the first and second trimesters, respectively. The stage and duration of infection were likely responsible for the severe clinical presentation and destruction of neurons, and, consequently, the depopulation of neurons in large areas of the cerebral cortex. This study provides strong evidence that ZIKV was the causative agent of microcephaly and other severe congenital malformations in the patients, resulting in fetal and neonatal deaths, as well as miscarriage.

## Figures and Tables

**Table 1:** Information about the 12 fatal cases of Zika virus (ZIKV) infection.

Case	State	Category	Lifetime	Sex	Date Birth (m/d/y)	Date Death (m/d/y)	Gestational Age	Maternal DOS	Maternal Symptoms	Case Information	Material
1	ES	Fetus (Miscarriage)	NA	NA	12/24/2015	12/24/2015	8 weeks	No symptoms	No symptoms	Ultrasound (8 weeks) detected retained dead fetus. Fetal skull disproportionately smaller than the trunk	fetus, placenta
2	RN	Stillbirth	NA	M	01/12/2016	01/12/2016	34 weeks (Preterm)	Unknown	Unknown	Macroscopic aspects: brain malformed, dilated lateral ventricles and the absence of the cerebellum; absence of nasal bone, clubfoot; bilateral cubital fold; absence of scrotum; hypoplastic penis; bilateral cryptorchidism	brain, liver, kidney, lung
3	PE	Stillbirth	NA	F	12/16/2015	12/16/2015	40 weeks (At term)	Unknown	Urinary tract infection treated during pregnancy	Microcephaly detected at birth	brain, liver, spleen, heart, kidney, lung, placenta
4	RN	Newborn	2 h	M	11/14/2015	11/14/2015	Unknown	First trimester	Symptoms suggestive of Zika infection	Newborn with microcephaly, large anterior fontanel, nasopalatine cleft, low-set ears, ginodactyly, clubfoot	brain, liver, kidney, lung
5	RN	Newborn	6 h	F	11/17/2015	11/17/2015	40 weeks (At term)	Before confirmation of pregnancy	General symptoms of viral disease and rash	Microcephaly with ventriculomegaly, arthrogryposis, pulmonary hypoplasia, atrial septal defect	brain, liver, kidney, lung
6	RN	Newborn	14 h	M	01/07/2016	01/07/2016	Unknown	Unknown	Unknown	Newborn with microcephaly (CP = 32 cm) and hydrocephalus, arthrogryposis. Macroscopic pulmonary hypoplasia, reduced brain tissue, cerebellar hypoplasia, abdominal testis, thoracolumbar scoliosis	brain, liver, kidney, lung
7	CE	Newborn	1 day	M	12/16/2015	12/17/2015	Unknown	Unknown	Unknown	Microcephaly	brain, liver, spleen, heart, kidney, lung, placenta, umbilical cord
8	CE	Newborn	2 days	M	12/30/2015	12/31/2015	Unknown	Unknown	Unknown	Microcephaly	brain, liver, spleen, heart, kidney, lung
9	PB	Newborn	1 day	M	12/26/2015	12/26/2015	28 weeks (Preterm)	First trimester	Fever and rash	Ultrasound (22 weeks) detected microcephaly, clubfoot, agenesis of fingers. Newborn with microcephaly (CP = 21 cm) and deformities of the hands and feet were observed. Macroscopy: decreased lungs and brain; lung, liver, pancreas, spleen and gut with a wine-like appearance	brain, liver, spleen, heart, kidney, lung
10	CE	Newborn	5 min	F	11/18/2015	11/18/2015	29 weeks (Preterm)	First trimester	Fever, myalgia, joint pain, and rash	Newborn with microcephaly and arthrogryposis, and possible esophageal atresia	blood, CSF, urine, brain, liver, spleen, thymus, heart, kidney, lung
11	CE	Newborn	1 day	F	12/16/2015	12/17/2015	36 weeks (Preterm)	Unknown	Unknown	Cesarean delivery for breech presentation, with the presence of meconium. Detected microcephaly (CP = 31 cm) and arthrogryposis. X-ray shows right diaphragmatic hernia	brain, liver, spleen, heart, kidney, lung
12	CE	Newborn	1 day	F	12/22/2015	12/22/2015	At term	Second trimester	Fever and rash	Detected microcephaly (CP = 30 cm) and bilateral ventriculomegaly on 35 weeks of gestational age.	serum, placenta

DOS: date of onset of symptoms; NA: not applicable; CSF: cerebrospinal fluid; ES: Espírito Santo State; RN: Rio Grande do Norte State; M: Male; F: Femal; CP: cephalic perimeter; PE: Pernambuco State; CE: Ceará State; PB: Paraíba State.

**Table 2:** Results of Histopathology, Immunohistochemistry (IHC) and Real Time PCR (RT-qPCR) in the 12 fatal cases of ZIKV infection.

Case	Category	Gestational Age	Lifetime	Material	Results		
					Histopathology	IHC	RT-qPCR
1	Fetus (Miscarriage)	8 weeks	NA	Fetus	Fetal tissue inside the yolk sac with necrotic lesions, but no observed inflammatory reaction	Pos	NR
				Placenta	Membrane and deciduous with necrosis, neutrophils and mononuclear cells. Areas with hyaline, mononuclear infiltrate, calcification foci, and abundant fibrinoid material	Pos	NR
2	Stillbirth	34 weeks (preterm)	NA	Brain	Tissue edema, vasocongestion in meninges. Nervous tissue with vascular proliferation, necrosis or apoptosis, gliosis, calcification multiple, edema. There are few neurons	Pos	Pos
				Liver	Autolysis	Pos	Pos
				Kidney	Vasocongestion. Renal tubes with autolysis	Pos	Pos
				Lung	Lung immaturity, presence of keratinized cells in alveolar spaces with amniotic fluid, and other areas with atelectasis; vessels with thickened walls	Pos	Pos
3	Stillbirth	40 weeks (At term)	NA	Brain	Edema and diffuse vasocongestion, neuronal apoptosis	Pos	NR
				Liver	Intense sinusoidal vasocongestion, macrovacuolar steatosis, little inflammatory infiltration of macrophages and plasma cells in portal tract.	Pos	NR
				Spleen	Vasocongestion	Neg	NR
				Heart	No changes	Neg	NR
				Kidney	Intense vasocongestion	Pos	NR
				Lung	Vasocongestion, presence of amniotic fluid and meconium in alveoli, bronchi, and bronchioles, suggestive of fetal distress	Neg	NR
				Placenta	Membranes preserved with multiple foci of calcification, fibrin, and vasocongestion	Neg	NR
4	Newborn	Unknown	2 h	Brain	Structure preserved but with edema and vasocongestion.	Pos	Neg
				Liver	Intense vasocongestion and focal erythropoiesis. Focal microvacuolar steatosis in some hepatocytes. No portal inflammatory reaction	Pos	Pos
				Kidney	Diffuse vasocongestion and renal pelvis bleeding	Pos	Neg
				Lung	Structure preserved with few areas of alveolar bleeding and vasocongestion	Neg	Neg
5	Newborn	40 weeks (At term)	6 h	Brain	Edema, vasocongestion, degenerative neural lesions (vacuolization of nucleus)	Pos	Pos
				Liver	Subcapsular bleeding, hepatocyte edema, macro and microvacuolar steatosis, inflammatory infiltrate in portal area and sinusoids	Neg	Neg
				Kidney	Glomerular immaturity, hemorrhagic foci and vasocongestion	Neg	Neg
				Lung	Interstitial pneumonia, alveolar bleeding and intense vasocongestion; hemorrhagic pleuritis	Neg	Neg
6	Newborn	Unknown	14 h	Brain	Necrosis, calcification, gliosis, edema, neuroapoptosis and intense vasocongestion in the cortical layer; perivascular lymphocytic infiltrate; dilatation of ventricles (ventriculomegaly)	Pos	Pos
				Liver	Hepatocytes with discreet and sparse macrovesicular steatosis. Sinusoidal intense vasocongestion. No portal inflammatory reaction	Neg	Neg
				Kidney	Vasocongestion	Neg	Neg
				Lung	Edema, alveoli bleeding, and vasocongestion	Pos	Neg
7	Newborn	Unknown	1 day	Brain	Meningeal thickening with vascularization intense, hyperemia, edema, lymphocytic inflammatory infiltrate; nervous tissue with edema, perivascular lymphocytic infiltrate, vasocongestion intense, and neuronal necrosis	Pos	Pos
				Liver	Erythropoiesis and sinusoidal congestion	Neg	Pos
				Spleen	Vasocongestion	Neg	Pos
				Heart	No changes	Neg	Pos
				Kidney	Diffuse vascular congestion	Neg	Pos
				Lung	Vasocongestion, alveolar collapse; Bronchi and interstitial septa with lymphocytic infiltrate	Neg	Pos
8	Newborn	Unknown	2 days	Placenta	No changes. Maturity corresponding to 3rd trimester of pregnancy	Neg	NR
				Umbilical cord	Normal aspect	Neg	NR
				Brain	Edema, vasocongestion and vascular proliferation, neuronal necrosis, focal depopulation, and calcifications	Pos	Neg
				Liver	Vasocongestion, hepatic steatosis macro and microvacuolar, erythropoiesis foci; inflammatory reaction in portal tract	Pos	Neg
				Spleen	Vasocongestion	Pos	Neg
				Heart	Muscle fibers with vacuolated aspect	Pos	Neg
				Kidney	Vasocongestion intense, predominantly in medullar area	Pos	Neg
				Lung	Vasocongestion, alveoli containing granular and acidophilus material, and desquamated epithelial cells, suggesting amniotic fluid aspiration and fetal distress	Pos	Neg
9	Newborn	28 weeks (preterm)	1 day	Brain	Multiple calcifications in cortical layer. Edema, necrosis, vascular proliferation, gliosis foci, neuronal depopulation, and residual neurons with necrosis.	Pos	Pos
				Liver	Focal erythropoiesis; partial autolysis of hepatocytes	Pos	Neg
				Spleen	Autolysis	Neg	Neg
				Kidney	No changes	Pos	Pos
				Lung	Large areas collapsed with acidophilus material brown-dark color, suggesting amniotic fluid and meconium aspiration and fetal distress	Neg	Pos
10	Newborn	29 weeks (preterm)	5 min	Blood	NA	NA	Neg
				CSF	NA	NA	Pos
				Urine	NA	NA	Neg
				Brain	NR	NR	Pos
				Liver	NR	NR	Neg
				Spleen	NR	NR	Neg
				Thymus	NR	NR	Neg
				Heart	NR	NR	Pos
				Kidney	NR	NR	Pos
				Lung	NR	NR	Pos
11	Newborn	36 weeks (preterm)	1 day	Brain	Meningeal thickening with abundant vascularization, vasocongestion, mononuclear infiltrate, edema. Cortical with neuronal depopulation, disorganization, neuronal satelitosis, focal gliosis, vascular proliferation, hyperemia, and intense edema	Pos	Pos
				Liver	Sinusoidal congestion and edema of hepatocytes	Pos	Pos
				Spleen	Vasocongestion	Neg	Pos
				Heart	No changes	Pos	Pos
				Kidney	Vasocongestion	Pos	Pos
				Lung	Vasocongestion	Neg	Pos
12	Newborn	At term	1 day	Serum	NA	NA	Pos
				Placenta	Membranes preserved. Chorionic villi vascularized with calcification foci	Pos	Pos

NA: not applicable; IHC: Immunohistochemistry; RT-qPCR: reverse transcription real-time polymerase chain reaction; NR: sample not obtained; CSF: cerebrospinal fluid; Pos: Positive; Neg: Negative.
